# After-Care Problems

**Published:** 1907-01

**Authors:** 


### AFTER-CARE PROBLEMS.

No movement in modern preventive medicine is more interesting or more full of promise than the awakening of attention in recent years to the "after-care" of individuals who have suffered from more or less chronic ailments and who, after treatment for some time in institutions, have been discharged as cured. Tuberculosis and insanity in particular have deservedly been the subjects of this special attention and any auxiliary that will reduce the morbidity and mortality for either of them must be heartily welcomed. The decrease of both of these affections is dependent to a much larger extent on the after-care of those who have been under special treatment than was imagined until a few years ago when the crying need of post-sanitarium measures at last found responsive auditors among the medical profession and those charitably inclined.

With regard to tuberculosis, no phase of the crusade against the disease is more important than proper provision for the socalled cured patients in order to prevent relapse. If a person goes back to the work and the environment in which he originally contracted tuberculosis, relapse is almost inevitable. This might have been so concluded on theoretic grounds, but experience has demonstrated it beyond all doubt since the establishment of sanatoria for the disease. Cured patients must not, as a rule, return to live the strenuous life of the large city, lest the period of latency of their tuberculosis—for radical cure is almost out of the question—will be at best but short. A country life without too much work and with plentiful opportunities for outdoor air and abundant food are the ideals of the post-sanitarium life of the tuberculosus. Unfortunately, these can not always be obtained and, if a decided improvement in this matter is to be brought abouæt, organization of the after-cure of the tuberculosis is as important as corresponding organization for the erection and maintenance of sanatoria and for the education of the masses ,with regard to the prophylaxis and proper management of the disease.

Fortunately, the after-care movement with regard to the other great series of affections which are just now rousing so much attention all over the world—those grouped under the name insanity—is already well under way and promises to advance rapidly. The first of these societies for the after-care of the insane was founded only a few years ago in England. Its methods are eminently practical. The secretary of the society visits the asylums, works in close co-operation with the medical superintendents and is notified by them when patients who are to be discharged cured are poor and without homes or friends. For such individuals boarding places—in the country for the woman, in the city for the men—have previously been arranged by the society. These are not large institutions or buildings owned by the society, for, as the first report of the committee on After-Care of the Insane of the State Charities Aid Association of New York insists, the society does not wish them, but prefers small cottage homes—small boarding houses as we should call them—where a man and his wife are willing to board these after-care patients. Their board is paid by the society usually for from one to six weeks until health is fully re-established and the individuals are able to work. The women require much more looking after than the men. Employment is found for both sexes so far as possible in a suitable environment, and then the society keeps in communication with them until they are absorbed into the community as ordinary self-supporting, self-respecting men and women.

It is almost needless to say that such an organization accomplishes work of the greatest possible utility and by preventing relapses decreases greatly the expense incident to the support of patients in the insane asylums, for relapsed cases often prove obstinately intractable and further improvement to an extent that will allow them to leave the asylum can scarcely be expected. An organization of a similar kind to that in England has been established during the present year in America and it seems only proper that the medical profession should encourage the good work that it is doing in every possible way. If corresponding organizations for the after-care of tuberculosis patients were to be established they too would doubtless accomplish most satisfactory work. If the community owes it to itself and its citizens that it shall take care of persons ailing from such diseases it would seem to be even more important that after their cure it should make every effort to maintain them in that state of improved health which its paternal care has secured for them.—*Ed. Jour. A. M. A.*, December 1st, 1906.

